# Redefining the Role of Langerhans Cells As Immune Regulators within the Skin

**DOI:** 10.3389/fimmu.2017.01941

**Published:** 2018-01-05

**Authors:** Heather C. West, Clare L. Bennett

**Affiliations:** ^1^Institute of Immunity and Transplantation, University College London, London, United Kingdom; ^2^Division of Cancer Studies, University College London, London, United Kingdom

**Keywords:** Langerhans cells, skin, epidermis, macrophages, migration

## Abstract

Langerhans cells (LC) are a unique population of tissue-resident macrophages that form a network of cells across the epidermis of the skin, but which have the ability to migrate from the epidermis to draining lymph nodes (LN). Their location at the skin barrier suggests a key role as immune sentinels. However, despite decades of research, the role of LC in skin immunity is unclear; ablation of LC results in neither fatal susceptibility to skin infection nor overt autoimmunity due to lack of immune regulation. Our understanding of immune processes has traditionally been centered on secondary lymphoid organs as sites of lymphocyte priming and differentiation, which is exemplified by LC, initially defined as a paradigm for tissue dendritic cells that migrate to draining LN on maturation. But, more recently, an awareness of the importance of the tissue environment in shaping effector immunity has emerged. In this mini-review, we discuss whether our lack of understanding of LC function stems from our lymph node-centric view of these cells, and question whether a focus on LC as immune regulators *in situ* in the skin may reveal clearer answers about their function in cutaneous immunology.

## Introduction

Langerhans cells (LC) are a unique population of mononuclear phagocytes that are seeded from common macrophage precursors in the skin epidermis before birth (1) (and reviewed in this topic). They are highly conserved across vertebrate species (2, 3) and this, with their location at the interface with the environment, suggests the strategic importance of LC as immune sentinels at the skin barrier surface.

Traditionally, immunologists have focused on secondary lymphoid organs as the center of T cell immunity, assuming that instructions given during the priming of naïve T cells by migratory and resident dendritic cells (DC) were sufficient for differentiation and effector function by T cells recruited to peripheral sites of tissue injury. However, the field is beginning to appreciate importance of the tissue environment in regulating effector and regulatory T cell function, and it is clear that antigen-presenting cell-T cell interactions play a key role in T cell survival and function outside lymphoid organs (4). Despite sharing an origin with other tissue-resident macrophages, differentiation of LC is associated with the acquisition of DC-like functions, namely the ability to migrate to skin-draining lymph nodes (LN) and interact with naïve T cells. Observation of this property in the 1980s has dominated the field, and as a result, studies to define LC function in the skin have focused largely on their role as DC-like cells in priming T cell immunity [reviewed by Romani et al. (5)]. However, to date, a consistent role for LC as primers of T cell immunity has not emerged. In particular, there are few scenarios, if any, in which removal of LC ablates immunity to infection, or results in the development of severe autoimmunity in mice; and, despite the common observation that LC are sufficient to prime T cell immunity after the experimental provision of antigen and adjuvants that may favor LC activation and migration [e.g., Ref. (6)], few papers have explicitly identified a crucial role for migratory LC, and not dermal DC, under physiological conditions.

**Figure 1:**
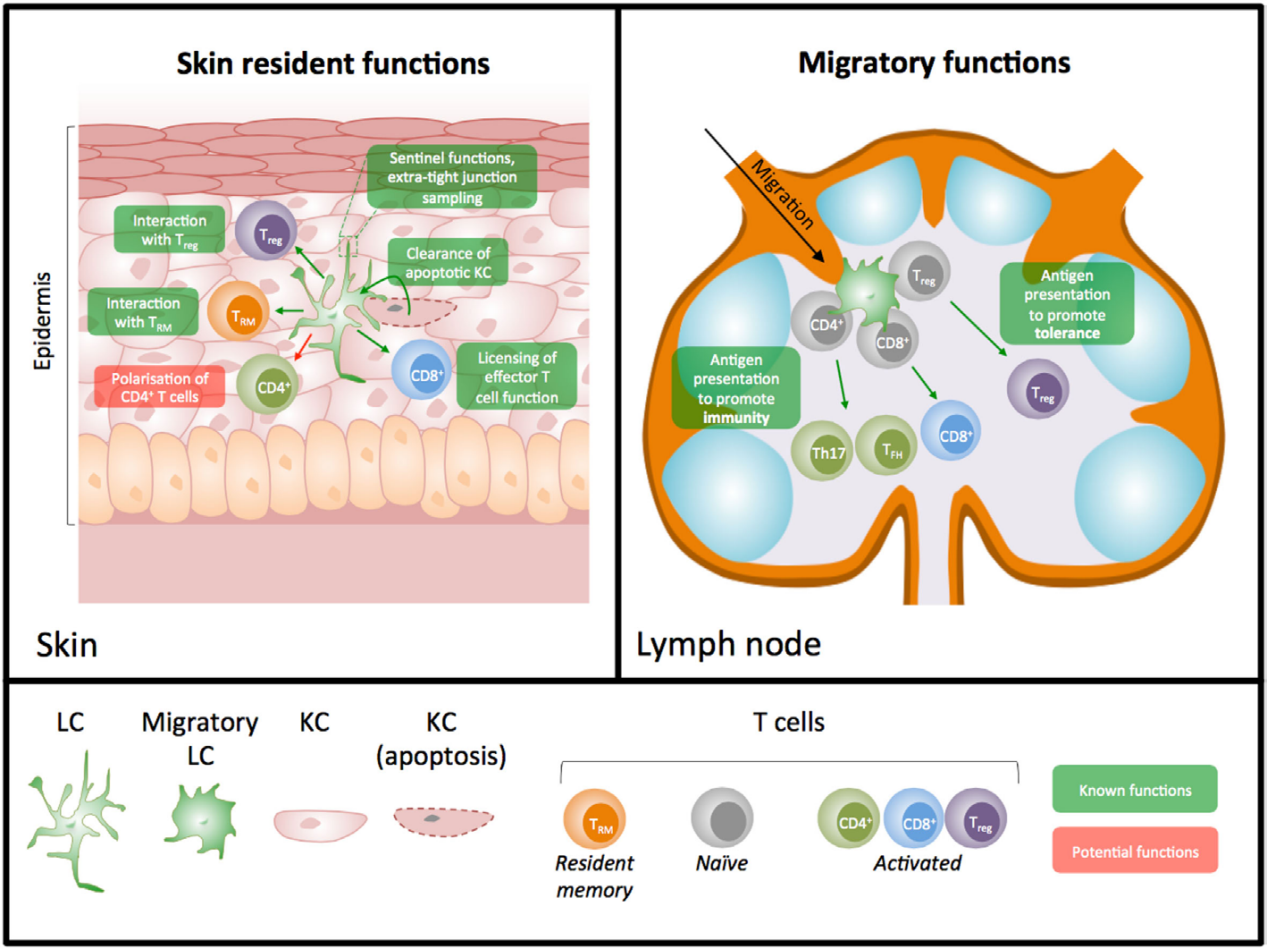
Diagram illustrating known and predicted skin resident versus migratory roles for Langerhans cells (LC). LC have important functions as resident cells in the skin and as migratory cells to the lymph nodes (LN). Studies have largely focused on their potential importance in priming T cell immunity in LN; however, it is now appreciated that LC have many functions *in situ* in the skin. Demonstrated functions (shown in green) in the skin include interaction with resident memory T cells, clearance of apoptotic keratinocytes, licensing of effector T cell function, sentinel functions, and interaction with regulatory T cells. Data further suggest the potential for other macrophage-like functions of LC such as a role in the polarization of CD4^+^ T cells *in situ* (shown in red).

We suggest that shifting our focus to potential roles for LC *in situ* in the skin, a function more in keeping with their development as macrophages, will provide clearer answers about the importance of these unique cells in skin immunity. In this mini-review, we will consider the evidence for LC functions within the skin (Figure 1), and discuss whether our historic focus on LC as exemplars of migrating DC has skewed our understanding of their role in skin immunity.

## Elucidating LC Function *In Situ* in the Skin

### Barrier Site Surveillance

#### Sensing the Local Environment

Mononuclear phagocytes have important functions within tissues, and barrier site non-migratory macrophages are essential for surveillance of the local environment. CX_3_CR_1_^+^ intestinal macrophages form dendritic projections, termed transepithelial dendrites, which penetrate the intestinal epithelium to sense commensal microbes and sample luminal antigens. Barrier integrity is maintained by the formation of tight junctions between the macrophages and epithelial cells (7, 8). In the central nervous system, microglia are highly dynamic in their resting state and rapidly extend and retract their processes (9, 10). This motility, termed synaptic pruning, allows them to make frequent and transient contact with synapses, actively engulfing synaptic material (11, 12), and is essential in nervous system development and maintenance (9, 13, 14).

Langerhans cells also constantly extend and retract dendrites between keratinocytes, in a behavior termed dendritic surveillance extension and retraction cycling habitude (15). Barrier integrity is maintained by the *de novo* formation of tight junctions between keratinocytes and LC, enabling LC to sample the extra-tight junction environment without loss of integrity (16). Thus, in this respect, LC can be seen to closely mimic non-migratory cells, leading to the question of how LC behavior compares to other resident macrophage populations once foreign material has been detected.

#### Local Interaction with Viruses

Langerhans cells are ideally positioned to respond to viruses that enter the body *via* the skin, namely human immunodeficiency virus (HIV), herpes viruses such as herpes simplex virus (HSV) or varicella zoster virus, and poxviruses (human papilloma virus, HPV), and this interaction has been extensively reviewed elsewhere [e.g., Ref. (17–19)]. LC express a number of pattern recognition receptors but do not efficiently internalize bacteria *in vitro*, leading to the suggestion that they preferentially prime antiviral immunity (20, 21). However, infection of LC by HSV or HPV leads to their destruction; priming of T cell immunity in this situation probably depends on transfer of viral antigen to other DC. In the case of HSV, non-infected epidermal LC may acquire antigen from apoptotic LC in the skin and migrate to draining LN. Here, transfer of antigen to cross-presenting DC subsets is required for the initiation of CD8^+^ T cell immunity (22, 23). By comparison, while LC appear to be the major infected immune cell after HIV infection *via* the anogenital tract (24), there is evidence that they may restrict HIV replication or transmission (25, 26), and their protective role in HIV infection continues to be investigated (27). In this respect, LC resemble other tissue macrophages, which also restrict HIV and other viral infections (28, 29). Thus, LC may provide a repository of virally infected cells under some conditions, but the implications for this interaction for innate and adaptive immunity still remain unclear.

### Innate Control of Skin Immune Homeostasis

A key role for macrophages as resident tissue cells is the maintenance of immune homeostasis. This occurs partly through their scavenger function, rapidly clearing debris from dying cells in the steady state and after infection. This uptake and recognition of apoptotic cells is an important mechanism for maintaining local immune tolerance to self-antigens (30, 31). TAM (TYRO3/AXL/MER) family receptor tyrosine kinases are expressed by macrophages, DC and natural killer cells in the immune system. Engagement of the receptors by their ligands, growth-arrest-specific (GAS)6, and protein S, enhances uptake of apoptotic cells and suppresses inflammation (32). In the gut, MER is upregulated by macrophages in response to induction of apoptosis in intraepithelial cells, probably to control local immune suppression to self-antigens (31). LC employ a similar innate mechanism to control local tolerance in the skin: apoptotic keratinocytes accumulate in the skin of mice depleted of LC (33); and transforming growth factor (TGF)-β induced expression of AXL by LC enhances uptake of GAS6-expressing apoptotic keratinocytes, inhibiting of production of inflammatory cytokines (34).

### Regulation of T Cell Function in the Skin

In addition to innate control of the immune environment, tissue-resident cells may also influence adaptive immunity by recruited regulatory and conventional T cells. The following section considers the evidence for LC mediating this function directly in the skin.

#### Enhancing the Accumulation and Function of Regulatory T Cells *In Situ*

Tissue macrophages play a key role in the suppression of local adaptive immunity, *via* direct and indirect interaction with CD4^+^ regulatory T cells (T_reg_). CX_3_CR_1_-expressing macrophages are an abundant population in the lamina propria of the small intestine, where they regulate T_reg_ differentiation *in situ* by production of IL-10, retinoid acid (RA) and TGF-β (35), and are critical for the local proliferation of T_reg_ and induction of oral tolerance (36). Likewise, lung-resident tissue macrophages constitutively express TGF-β and RA to induce T_reg_ within the lung tissue (37, 38), while IL-10 production by Kupffer cells in the liver, and microglia in the brain, has been implicated in the induction of T_reg_-induced tolerance (39–42). In addition to promoting cytokine-mediated differentiation and function, macrophages directly interact with T_reg_ *in situ*. In a model of experimental autoimmune encephalomyelitis, direct contacts between sialoadhesin^+^ macrophages and T_reg_ regulated local immune suppression to limit progression of disease (43), and in the liver, presentation of antigen and arrest of T_reg_ by Kupffer cells led to local secretion of IL-10 (41).

Langerhans cells have been directly implicated in a number of T_reg_-dependent models of immune suppression in the skin but, consistent with the LN-centric view of LC research, it is often unclear whether cellular interactions between LC and T_reg_ are occurring in the LN, the skin, or both. For example, while use of bone marrow chimeras in which radio-resistant LC are selectively deficient for the gene *Cdkn1a* suggests a clear role for LC in the priming of CD4^+^ T_reg_ in LN after exposure to ionizing radiation (44), the precise location(s) at which LC are required has not been defined for the accumulation of T_reg_ in the ear skin of *Leishmania*-infected mice (45), or expansion and function of activated ICOS^+^ T_reg_ in a murine model of skin sensitization and tolerance (46). However, cognate interaction between LC and CD4^+^ T cells inhibits effector T cell responses after challenge with topical sensitizers (47), and resting LC from human skin selectively and specifically induce the activation and proliferation of skin resident T_reg_ *in vitro* (48). Upregulation of receptor activator of nuclear factor κ B (RANK) ligand by apoptotic keratinocytes induces IL-10 production by LC (49), inferring the potential of LC to directly regulate effector T cell immunity *in situ*, after exposure to ultraviolet (UV)B radiation. This concept is supported by Loser et al. who demonstrated that UV-induced immunosuppression is mediated by RANK-RANKL signaling between LC and keratinocytes, resulting in an increased capacity of LC to induce IL-10-driven CD4^+^ T_reg_ proliferation (50). CD300a is a glycoprotein expressed by myeloid cells, which binds phosphatidylserine when exposed on the plasma membrane of apoptotic cells. Interaction between CD300a^+^ LC and apoptotic epithelial cells in the skin restricts numbers of local T_reg_, mimicking interactions between epithelial cells and CD11b^+^CX_3_CR_1_^+^ cells in the lungs and gut. This interaction was required for control of *S. typhimurium* in the gut, but promoted deleterious inflammatory responses including atopic dermatitis in the skin (51). Together, these data strongly support a tissue role for LC in controlling immune suppression by T_reg_.

Elegant *in vivo* imaging studies by the Germain lab recently revealed close localization of migrating dermal DC with T_reg_ clusters in mouse LN in the steady state (52), supporting a previous study that demonstrated a specific role for dermal RelB^+^ DC in the maintenance of skin tolerance (53). Given the sessile behavior of LC in the steady state (54), these data support a dominant role for dermal DC in maintaining day-to-day tolerance to skin antigens. Therefore, the challenge now is to understand whether there are contexts in which LC may take over this regulatory role in LN. A recent study attempted to address this question using a novel genetic model of inducible neo-antigen expression by LC, but not other Langerin^+^ DC, in the steady state (LCre-GFPOVA mice) (55). In this model, presentation of endogenous ovalbumin leads to priming of CD8^+^ T cells in LN and accumulation of cutaneous CD4^+^ T_reg_. It would be informative to test how the T_reg_ are primed in this model and whether LC are required for their suppressive function in the skin.

#### Activation of Effector T Cell Immunity in the Skin

While it is accepted that the tissue-immune environment is important for the differentiation and function of T_reg_, textbook immunology tells us that priming of naïve conventional T cells in LN provides all the requisite signals for differentiation into functional effector/memory T cells. However, numerous studies have now demonstrated the importance of interactions between myeloid cells and conventional T cells for T cell function and survival within peripheral tissues [reviewed in Ref. (4), see also Ref. (56)]. We have demonstrated a unique and novel role for LC in licensing effector function of CD8^+^ T cells in the epidermis (57). In this section, we will consider the evidence to suggest that LC may also drive the function of other effector T cells *in situ*.

##### Licensing of CD8^+^ T Cell Function by LC

Langerhans cells are highly radio-resistant and, as such, persist following conditioning of patients prior to allogeneic stem cell transplantation for blood cancers and other hematopoietic diseases. Entry of activated donor T cells into inflamed organs, including the skin, frequently leads to graft-versus-host disease (GVHD) in these patients (reviewed in this topic by Santos e Sousa and Chakraverty). LC are sufficient to prime the donor T cell response leading to GVHD (58), but are not required for systemic GVHD in the presence of conventional DC populations (59). To understand whether LC are important in cutaneous GVHD, we combined the Langerin-diphtheria toxin receptor model [in which LC are inducibly depleted upon injection of diphtheria toxin (60)] with a murine allogeneic model of GVHD (61). We demonstrated that LC were not required for priming of donor T cells, in agreement with the published literature (59). However, when we focused on the cellular interactions occurring in the skin, we observed that LC licensed the upregulation of epidermal effector CD8^+^ T cell function, leading to GVHD at the site of inflammation (57). This research, therefore, revealed a novel role for LC outside LN.

##### LC-Dependent Control of CD4^+^ T Cell Function

Polarized CD4^+^ T cells that exit LN after priming maintain the plasticity to adapt to the tissue environment at their destination (62). This is particularly true for Th17 cells that may acquire a different functional fate depending on signals received from their surroundings. IL-23 production by monocytes and macrophages in inflamed tissues has been closely linked to the activation of IL-17-producing CD4^+^ T cells [e.g., Ref. (63–65)], and Foucher et al. demonstrated that interaction between human macrophages and memory CD4^+^ T cells *via* membrane-bound IL-1α is sufficient to polarize T cells toward Th1 or Th17-like phenotypes (66). Moreover, “inflammatory DC” from patient synovial fluid or tumor ascites stimulate IL-17 production from autologous CD4^+^ T cells, suggesting that myeloid cells may directly induce differentiation of Th17 cells within tissues (67).

Based on these macrophage data, it seems likely that LC perform a similar role in the skin, and it is clear that activated LC produce Th17-polarizing cytokines *ex vivo* (68–70). However, the ability of LC to induce differentiation and activation of epidermal Th17 cells *in situ* has not been directly investigated. LC are required for the priming of Th17 cells in response to topical *Candida albicans* infection (71, 72), and for the accumulation of both IL-17^+^CD4^+^ αβ and γδ T cells in the epidermis of mice with *Staphylococcus aureus* dysbiosis (73); however, neither study investigated whether LC are required *in situ* for the functional activity of cutaneous Th17 cells. Likewise, there are currently conflicting data on whether IL-23 production by LC is important for IL-17-dependent psoriasis-like disease (74, 75), and, to date, studies have focused on an Imiquimod-driven, αβ T cell-independent, γδ T cell-dependent model of disease. Thus, the potential importance of interactions between LC and epidermal CD4^+^ T cells in psoriasis has not been established.

#### LC and Cutaneous-Resident Memory T Cells

Human skin contains billions of T cells, and the majority express markers associated with retention as resident memory cells (T_rm_) (76). The concept of LC eliciting rapid localized immunity *via* activation of these cells is, therefore, very attractive. Precedent for interaction between tissue macrophages and T_rm_ has been set in the vaginal mucosa, where macrophage-derived CCL5 is required for the recruitment and maintenance of clusters of CD4^+^ T_rm_ that protect against viral challenge (77). However, despite evidence that LC interact directly with CD4^+^ memory T cells in human skin (48), and data demonstrating that LC interact with, and may control local migration of T_rm_ within the epidermis (78), formation of CD8^+^ T_rm_ is not impaired in the absence of LC (79).

IL-17 production by human skin T_rm_ is associated with psoriasis (80). However, a subsequent study from the same lab demonstrated that incubation with IL-6, IL-1β, and IL-23 were not sufficient *in vitro* to induce IL-17 production by CD8^+^ T_rm_ from healthy skin, suggesting a requirement for cognate T cell receptor-mediated interactions *in situ* (81). These data strongly infer a role for local interactions with LC, or other epidermal cells, in controlling T_rm_ fate in the context of autoimmunity.

## Concluding Remarks: Why Do LC Evolve the Capacity to Migrate?

Despite the development of LC from a common macrophage precursor (1), transcriptional profiling demonstrates a gene expression profile that is more similar to migrating DC than other macrophage populations (3, 82), and LC share an antigen processing and presentation machinery with DC (83). Resident macrophage precursors that are recruited into different sites respond to tissue-specific signals and differentiate into a functional cell type that is adapted to that niche (84). For example, lung macrophages develop the capacity to phagocytose excessive surfactant proteins, thereby preventing alveolar proteinosis (85). These observations lead us to anticipate the existence of environmental pressures in the epidermis leading to functional evolution of LC toward a DC-like cell. However, given the paucity of data showing a clear requirement for LC in priming immunity to infections, and the frequency of DC in the dermis, the unanswered question remains of why the acquisition of a DC-like function by LC is so important for protection of the organism?

Egress of LC from the keratinocyte network in the epidermis to LN is a carefully choreographed process. In the epidermis, inflammation and/or pathogen-derived signals block TGF-β-mediated retention by integrins (79, 86) and promote downregulation of the cell adhesion protein E-cadherin (87), together allowing release of LC from surrounding keratinocytes. Activated LC then secrete metalloproteinases (MMP) 2 and 9, required to break though the basement membrane and enter the dermis (88). Once in the dermis, LC upregulate the chemokine receptor CCR7 that conducts entry into the dermal lymphatics (89, 90), mirroring the migratory pathway used by tissue DC to enter LN.

The requirement to extricate themselves from the keratinocyte network in the epidermis means that LC are slower to arrive in draining LN than dermal DC after antigenic challenge, suggesting that dermal DC may dominate in priming T cell responses to antigens that enter the dermis (54, 91). However, there are scenarios in which LC appear to take charge. Epicutaneous infection models with the pathogens *C. albicans* or *S. aureus* have demonstrated clear roles for LC in priming protective CD4^+^ T cell responses (71, 72, 92), and ionizing radiation specifically activates LC to prime T_reg_ in LN (44). Human, but not murine, LC express high levels of the invariant MHC-like molecule, CD1a. Recognition of CD1a on LC by recruited autoreactive T cells, or T cells specific for the poison ivy lipid urushiol, results in enhanced activation of IL-22- or IL-17-producing CD4^+^ T cells, respectively (93, 94). These data support murine studies suggesting that LC prime CD4^+^ T cells responses to some topically applied sensitizers that do not reach the dermis (95, 96), potentially demonstrating a requirement for migratory epidermal cells in the rapid initiation of T cell responses to topical sensitizers or irritants.

Langerhans cells have also emerged as key players in the activation of CD4^+^ T follicular helper cells (T_fh_), which are required for germinal center formation and antibody affinity maturation in response to infection or vaccination (97). Here, ablation of LC results in clear defects in the formation of germinal centers and production of mature antibodies (92, 98). Importantly, this requirement for LC persists when antigen is delivered into the dermis and, therefore, should theoretically be preferentially acquired by dermal DC (97, 99). By contrast, an alternative study demonstrated that loss of LC led to increased production of autoantibodies in a murine model of lupus dermatitis (100), implying that, under steady state conditions, LC may also regulate activation of T_fh_, either directly or indirectly *via* T_reg_. However, dermal DC also prime T_fh_ (101), indicating that the need to generate antibody responses to skin antigens is not a sufficient functional pressure to explain the acquisition of migratory function by LC.

In conclusion we argue that, while significant progress has been made in our understanding of the key role LC play in cutaneous immunity, we now need to shift our focus from LN to the skin. Precise definition of the sites of interaction between LC and T cells, or other cells, is needed to determine the selective pressures that drive the relative tissue-resident or migratory DC-like functions of these unique cells.

## Author Contributions

HW and CB formulated opinions and concepts for the mini-review and wrote it together.

## Conflict of Interest Statement

The authors declare that the research was conducted in the absence of any commercial or financial relationships that could be construed as a potential conflict of interest.
